# Immune Checkpoint Inhibitor‐Based Therapy as the First‐Line Treatment for Advanced Non‐Small Cell Lung Cancer: Efficacy, Challenges, and Future Perspectives

**DOI:** 10.1111/1759-7714.70113

**Published:** 2025-06-23

**Authors:** Xingxiang Pu, Yu Zhou, Jingyi Wang, Lin Wu

**Affiliations:** ^1^ Department of Medical Oncology, Lung Cancer and Gastrointestinal Unit The Affiliated Cancer Hospital of Xiangya School of Medicine, Central South University/Hunan Cancer Hospital Changsha China; ^2^ The Second Department of Thoracic Oncology The Affiliated Cancer Hospital of Xiangya School of Medicine, Central South University/Hunan Cancer Hospital Changsha China; ^3^ Xiangya Lung Cancer Center, Xiangya Hospital, Central South University Changsha China

**Keywords:** first‐line treatment, immune checkpoint inhibitor, NSCLC, PD‐L1

## Abstract

The selection of initial systemic treatment for advanced non‐small cell lung cancer (NSCLC) depends on histological subtypes, oncogenic driver identification through genomic profiling, and programmed death‐ligand 1 (PD‐L1) expression quantification. The choice of first‐line treatment is crucial as patients with advanced NSCLC may not have the opportunity to receive second‐ or later‐line therapies due to the rapid progression of the disease. Current guidelines recommend pretreatment PD‐L1 expression quantification evaluation prior to initiating systemic therapy in advanced NSCLC. Except for histology, PD‐L1 expression, and absence of actionable driver mutations, single‐agent immune checkpoint inhibitors (ICIs) are foundational first‐line interventions. ICIs combined with chemotherapy or other ICIs have shown improved survival outcomes compared to ICI monotherapy. However, choosing the best option can be challenging due to limited head‐to‐head comparisons. Treatment decisions are often influenced by drug availability, reimbursement coverage, and patient's economic conditions. Despite the development of new ICI therapies, overall survival data seem to have plateaued, highlighting the need for sustained investigations and extensive clinical validation studies to develop novel therapies, optimize ICI combinations, and monitor adverse effects. Collaboration among data scientists, clinicians, biologists, and policymakers is essential to establish biomarkers that enhance patient selection and overall survival in NSCLC.

## Graphical abstract

Immune checkpoint inhibitor‐based first‐line therapy improves survival in advanced NSCLC, yet its efficacy is moderated by PD‐L1 expression and genomic profiles. Real‐world challenges, including limited comparative data and accessibility disparities, demand multidisciplinary collaboration to explore biomarkers, develop novel treatment combinations, and make policy optimization, which would ultimately transcend the current therapeutic plateau.

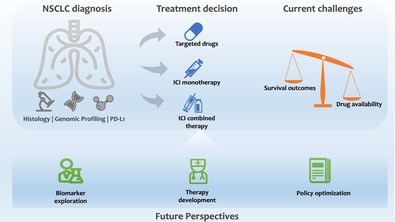

## 1 Introduction

Lung cancer stands as the leading etiology of cancer‐attributable mortality globally. Recent epidemiological surveillance data indicate approximately 2.2 million incident cases and 1.8 million cancer‐associated deaths were recorded in 2020 [1]. Lung cancer has been associated with environmental and lifestyle factors, of which cigarette smoking is the most important and responsible for approximately 80%–90% of lung cancer deaths [2, 3]. In the United States, the 5‐year relative survival rate for lung cancer is reported at 25.4%, while in China, it is slightly higher at 26.5% [4, 5]. Notably, over half of patients were with advanced stage non‐small cell lung cancer (NSCLC), which is associated with a significantly lower 5‐year relative survival rate of 8.2%. To facilitate earlier detection, early screening is advised for high‐risk individuals [6]. NSCLC represents the most common type of lung cancer, constituting about 85% of all lung cancer [7].

Before the presence of targeted therapies and immunotherapy, the sole treatment available for advanced NSCLC was platinum‐based chemotherapy [8]. During the last 20 years, significant progression in the treatment of NSCLC has enhanced the comprehension of the biological characteristics of the disease and the underlying mechanisms driving tumor advancement [9, 10]. Presently, the therapeutic approach for advanced NSCLC is determined by the histological subtype of the cancer and the existence of specific biomarkers [11].

In patients who do not harbor actionable oncogenic drivers, such as ALK rearrangements, BRAF mutations, EGFR mutations, or ROS1 rearrangements, existing clinical guidelines advocate for the use of immune checkpoint inhibitors (ICIs), either as monotherapy or in combination with chemotherapy, as the primary treatment modality [12]. Given the dynamic and swiftly evolving landscape of ICI‐based therapeutic strategies, numerous pertinent inquiries remain to be addressed, including the optimization of combination therapy approaches and the identification of relevant biomarkers. Furthermore, the advent of novel therapeutic targets introduces additional avenues for treatment in NSCLC. This review aims to systematically delineate the current landscape and future directions of ICI‐based therapies as a first‐line intervention for advanced NSCLC, thereby offering valuable insights to inform forthcoming clinical investigations.

## 2 Mechanism of Action of ICIs

**FIGURE 1:**
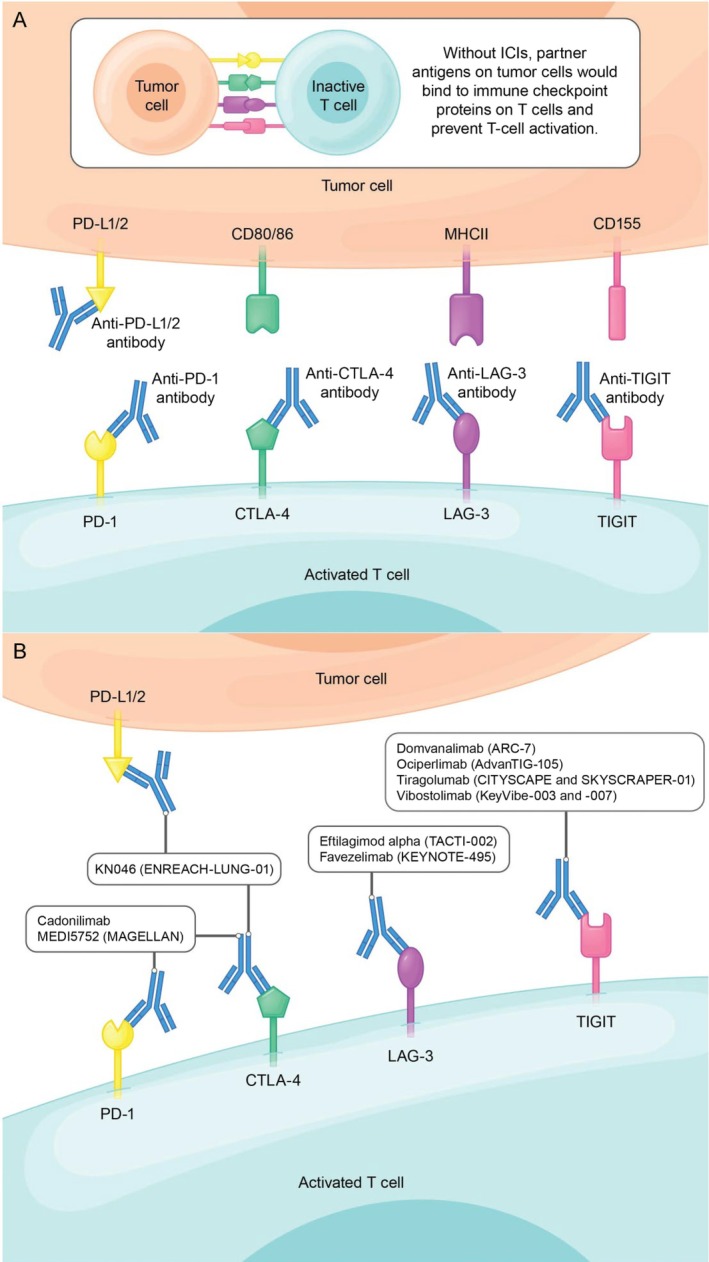
ICIs allow T cells to stay activated by preventing immune checkpoint receptors on T cells from binding to their partner antigens on tumor cells. (A) Schematic of ICI mechanism of action with current ICIs for NSCLC. (B) Novel ICI therapies being investigated in advanced NSCLC (with their corresponding clinical trials). KN046, cadonilimab, and MEDI5752 are bispecific antibodies. CD, clusters of differentiation; CTLA‐4, cytotoxic T‐lymphocyte associated protein 4; ICI, immune checkpoint inhibitor; LAG‐3, lymphocyte activation gene 3 protein; MHC, major histocompatibility complex; NSCLC, non‐small cell lung cancer; PD‐1, programmed cell death protein 1; PD‐L1/2, programmed cell death 1 ligand 1/2; TIGIT, T‐cell immunoreceptor with Ig and ITIM domains.

Immune checkpoints refer to specific proteins located on the surface of T cells that can be recognized and interact with corresponding antigens presented by tumor cells and other immune cells [13]. Upon the binding of immune checkpoints to their partner antigens, a signal is transmitted that functions similarly to a brake, resulting in the deactivation of T cells and a subsequent decline in immune functionality. ICIs represent a category of immunotherapy agents that operate by attaching to immune checkpoint proteins, thereby obstructing their interaction with partner antigens. The molecular intervention obstructs programmed cell death protein 1 (PD‐1)/programmed death‐ligand 1 (PD‐L1) axis‐mediated coinhibitory signaling that normally maintains immunological tolerance, consequently reversing T cell exhaustion phenotypes and augmenting lymphocyte‐mediated tumoricidal activity within the tumor microenvironment (Figure 1A). Numerous immune checkpoints play pivotal roles in this intricate process; however, the most substantiated candidates for ICI targeting include PD‐1, PD‐L1, and cytotoxic T‐lymphocyte‐associated protein 4 (CTLA‐4) [14]. PD‐1 is present on T cell surfaces and interacts with PD‐L1 and PD‐L2. While PD‐L1 is broadly expressed in inflamed tissues and on malignant cells, PD‐L2 predominantly appears in antigen‐presenting cells [15]. The functional interplay between PD‐1 and PD‐L1 is crucial for modulating immune responses and preserving immune homeostasis. The interaction of PD‐L1 with PD‐1 triggers an internal regulatory mechanism that inhibits T‐cell activation, thereby thwarting the ability of T cells to attack and eliminate tumor cells. Elevated levels of PD‐L1 expression can facilitate immune evasion, wherein tumor cells manipulate immune cells to escape immune detection [14]. Notably, in NSCLC, PD‐L1 is detected in 35%–95% of tumor biopsies, potentially contributing to the immune evasion frequently observed in lung cancer cases [16]. Additionally, lower PD‐L1 expression showed association with reduced survival rates and a poor prognosis [14].

CTLA‐4 serves as a ligand functioning as an immunomodulator in the interplay between tumor cells and immune cells; it is notably expressed in activated T cells to inhibit subsequent activation [14]. A specific domain within CTLA‐4 conveys inhibitory signals to T cells, thereby diminishing the immune response, which facilitates the proliferation and dissemination of tumor cells. Focusing on these pathways represents a promising approach to address the difficulties encountered in treating advanced NSCLC.

## 3 Clinical Studies of Anti‐PD‐1/PD‐L1/CTLA‐4 Antibodies

### 3.1 First‐Line Monotherapy Clinical Trials

At present, the initial treatment strategy for patients with advanced NSCLC involves the administration of single‐agent therapies. This recommendation applies universally, irrespective of histological classification, when the PD‐L1 expression levels are ≥ 50%, and in the absence of actionable driver mutations as determined by testing (Table 1).

**TABLE 1:** Efficacy of ICI monotherapies for the treatment of advanced NSCLC regardless of histology.

Drug	Comparator	Study (NCT #)	ORR, %	DOR, median, months	PFS		OS	
					Median, months	HR (95% CI)	Median, months	HR (95% CI)
Pembrolizumab (*n* = 154)	Chemotherapy (*n* = 151)	KEYNOTE‐024 [17, 18] (NCT02142738)	44.8 vs. 27.8	NR vs. 6.3	10.3 vs. 6.0	0.50 (0.37–0.68)	30.0 vs. 14.2	0.63 (0.47–0.86)
Pembrolizumab (*n* = 299)	Chemotherapy (*n* = 300)	KEYNOTE‐042 [19] (NCT02220894)	39 vs. 32	20.2 vs. 10.8	7.1 vs. 6.4	0.81 (0.67–0.99)	20.0 vs. 12.2	0.69 (0.56–0.85)
Atezolizumab (*n* = 107)	Chemotherapy (*n* = 98)	IMpower110 [20] (NCT02409342)	38.3 vs. 28.6	NE	8.1 vs. 5.0	0.63 (0.45–0.88)	20.2 vs. 13.1	0.59 (0.40–0.89)
Cemiplimab (*n* = 283)	Chemotherapy (*n* = 280)	EMPOWER‐Lung 1 [21] (NCT03088540)	39 vs. 20	16.7 vs. 6.0	8.2 vs. 5.7	0.54 (0.43–0.68)	NR vs. 14.2	0.57 (0.42–0.77)

Abbreviations: CI, confidence interval; DOR, duration of response; HR, hazard ratio; ICI, immune checkpoint inhibitor; NCT, National Clinical Trial; NE, not evaluable; NR, not reached; NSCLC, non‐small cell lung cancer; ORR, objective response rate; OS, overall survival; PFS, progression‐free survival.

The PD‐1 inhibitor pembrolizumab has obtained regulatory approval for first‐line monotherapy in advanced NSCLC patients demonstrating ≥ 1% PD‐L1 tumor proportion score (TPS) [22]. This therapeutic indication derives from pivotal Phase III investigations, including KEYNOTE‐024 (NCT02142738) and KEYNOTE‐042 (NCT02220894) [17, 18, 19, 23]. KEYNOTE‐024 prospectively randomized treatment‐naïve advanced NSCLC patients with PD‐L1 TPS ≥ 50% to either pembrolizumab (200 mg Q3W) or platinum‐doublet chemotherapy [17]. Superior clinical outcomes emerged in the immunotherapy arm: median PFS extended by 4.3 months (10.3 vs. 6.0 months), accompanied by elevated objective response rates (44.8% vs. 27.8%), prolonged median DOR (29.1 vs. 6.3 months), and reduced Grade ≥ 3 TRAEs (31.2% vs. 53.3%). Longitudinal follow‐up revealed durable survival benefits, with 5‐year OS rates doubling in the pembrolizumab cohort (31.9% vs. 16.3%) [23]. Subsequent KEYNOTE‐042 extended these observations to PD‐L1 TPS ≥ 1% populations, demonstrating statistically significant OS improvement with pembrolizumab vs. chemotherapy (HR = 0.81; 95% CI = 0.71–0.93; *p* = 0.0018) [19]. These cumulative findings prompted FDA label expansion in April 2019, authorizing pembrolizumab as primary therapy for metastatic NSCLC with PD‐L1 TPS ≥ 1% [24].

The PD‐L1‐targeting humanized IgG1 monoclonal antibody atezolizumab received accelerated FDA approval in May 2020 as first‐line therapy for advanced NSCLC with high PD‐L1 expression, based on groundbreaking outcomes from the Phase III IMpower110 trial (NCT02409342) [25]. This international multicenter randomized controlled study enrolled 572 treatment‐naïve metastatic NSCLC patients preselected for PD‐L1 positivity (tumor cell [TC] or immune cell [IC] ≥ 1% via VENTANA PD‐L1 [SP142] assay), randomly assigning them to either atezolizumab monotherapy (1200 mg q3w) or platinum‐based doublet chemotherapy [20]. Notably, among patients expressing the highest levels of PD‐L1, those receiving atezolizumab showed improved OS (20.2 vs. 13.1 months), enhanced PFS (8.1 vs. 5.0 months), and a higher response rate (38.3% vs. 28.6%) relative to the chemotherapy cohort. These statistically robust efficacy outcomes, combined with manageable safety profiles (Grades 3 and 4 TRAE 30.1% vs. 52.5%), culminated in the FDA's May 18, 2020 label update (BLA 761034 s15), establishing atezolizumab as the standard first‐line therapy for metastatic NSCLC patients exhibiting PD‐L1‐high expression (TC ≥ 50% or IC ≥ 10%) [26].

The anti‐PD‐1 monoclonal antibody cemiplimab was investigated as frontline monotherapy for advanced NSCLC with PD‐L1 TPS ≥ 50% in the global Phase III EMPOWER‐Lung 1 trial (NCT03088540) [20]. This open‐label randomized study allocated 710 treatment‐naïve patients (2:1 ratio) to receive either cemiplimab 350 mg q3w or platinum‐doublet chemotherapy. At a median follow‐up of 26.4 months, cemiplimab demonstrated unprecedented survival benefits: median OS was not reached vs. 14.2 months for chemotherapy (HR = 0.57; 95% CI = 0.42–0.77; *p* = 0.0002), with PFS extended by 2.5 months (8.2 vs. 5.7 months; HR = 0.54; 95% CI = 0.43–0.68; *p* < 0.0001). These practice‐changing results led to FDA priority review approval on February 22, 2021, establishing cemiplimab as a primary treatment option for PD‐L1‐high metastatic NSCLC [27].

Empirical evidence substantiates the therapeutic superiority of PD‐(L)1‐targeted biological agents as standalone interventions in treatment‐naïve NSCLC. While ICI monotherapies have significantly enhanced survival rates relative to chemotherapy in the initial treatment phase, there remains a necessity for additional enhancements. Consequently, ICI combination therapies were evaluated to potentially optimize outcomes across heterogeneous NSCLC populations, encompassing those who exhibit a lack of responsiveness to ICI monotherapies.

### 3.2 First‐Line Clinical Trials of ICIs Combined With Chemotherapy

The integration of ICIs, either in conjunction with another ICI or through alternative therapeutic approaches, holds significant promise for enhancing treatment efficacy while attenuating treatment toxicities [28]. The exploration of ICI combination treatment has been undertaken to assess their effectiveness through the simultaneous blockade of various targets. In the context of patients suffering from advanced NSCLC, multiple treatment regimens that amalgamate ICIs with chemotherapy have shown better efficacy compared to chemotherapy alone in the first‐line settings, as evidenced by Phase 3 clinical trials (Table 2). These ICIs include pembrolizumab, atezolizumab, camrelizumab, tislelizumab, sintilimab, sugemalimab, toripalimab, serplulimab, cemiplimab, and penpulimab.

**TABLE 2:** Efficacy of ICI combinations with chemotherapy for the treatment of advanced NSCLC.

Drug	Comparator	Study (NCT #)	Histology	ORR, %	DOR, median, months	PFS		OS	
						Median, months	HR (95% CI)	Median, months	HR (95% CI)
Pembrolizumab + chemotherapy (*n* = 410)	Placebo + chemotherapy (*n* = 206)	KEYNOTE‐189 [29] (NCT02578680)	Nonsquamous only	48.0 vs. 19.4	12.4 vs. 7.1	9.0 vs. 4.9	0.48 (0.40–0.58)	22.0 vs. 10.7	0.56 (0.45–0.70)
Pembrolizumab + chemotherapy (*n* = 278)	Placebo + chemotherapy (*n* = 281)	KEYNOTE‐407 [30] (NCT02775435)	Squamous only	62.6 vs. 38.4	8.8 vs. 4.9	8.0 vs. 5.1	0.57 (0.47–0.69)	17.1 vs. 11.6	0.71 (0.58–0.88)
Atezolizumab + chemotherapy (*n* = 451)	Chemotherapy (*n* = 228)	IMpower130 [31] (NCT02367781)	Nonsquamous only	49.2 vs. 31.9	8.4 vs. 6.1	7.0 vs. 5.5	0.64 (0.54–0.77)	18.6 vs. 13.9	0.79 (0.64–0.98)
Atezolizumab + bevacizumab + chemotherapy (*n* = 400)	Bevacizumab + CT (*n* = 400)	IMpower150 [32, 33] (NCT02366143)	Nonsquamous only	63.5 vs. 48.0	9.0 vs. 5.7	8.3 vs. 6.8	0.62 (0.52–0.74)	19.5 vs. 14.7	0.80 (0.67–0.95)
Toripalimab + chemotherapy (*n* = 309)	Placebo + chemotherapy (*n* = 156)	CHOICE‐01 [34] (NCT03856411)	Squamous and nonsquamous	65.7 vs. 46.2	8.4 vs. 4.2	8.4 vs. 5.6	0.49 (0.39–0.61)	NR vs. 17.1	0.69 (0.53–0.92)
Penpulimab + chemotherapy (*n* = 175)	Placebo + chemotherapy (*n* = 175)	AK105‐302 [35] (NCT03866993)	Squamous only	71.4 vs. 44.0	8.3 vs. 3.0	7.6 vs. 4.2	0.44 (0.34–0.56)	NR vs. 19.8	0.55 (0.40–0.75)
Cemiplimab + chemotherapy (*n* = 312)	Placebo + chemotherapy (*n* = 154)	EMPOWER‐Lung 3 [36, 37] (NCT03409614)	Squamous and nonsquamous	43.6 vs. 22.1	16.4 vs. 7.3	8.2 vs. 5.5	0.55 (0.44–0.68)	21.1 vs. 12.9	0.65 (0.51–0.82)
Serplulimab + chemotherapy (*n* = 358)	Placebo + chemotherapy (*n* = 179)	ASTRUM‐004 [38, 39] (NCT04033354)	Squamous only	NA	NA	NA	NA	NA	NA
Camrelizumab +chemotherapy (*n* = 205)	Chemotherapy (*n* = 207)	CameL [40] (NCT03134872)	Nonsquamous only	60.5 vs. 38.6	17.6 vs. 9.9	11.3 vs. 8.3	0.60 (0.45–0.79)	NR vs. 20.9	0.73 (0.53–1.02)
Camrelizumab + chemotherapy (*n* = 193)	Placebo + chemotherapy (*n* = 196)	CameL‐sq [41] (NCT03668496)	Squamous only	64.8 vs. 36.7	13.1 vs. 4.4	8.5 vs. 4.9	0.37 (0.29 –0.47)	NR vs. 14.5	0.55 (0.40–0.75)
Sintilimab + chemotherapy (*n* = 266)	Placebo + chemotherapy (*n* = 131)	ORIENT‐11 [42] (NCT03607539)	Nonsquamous only	51.9 vs. 29.8	NR vs. 5.5	8.9 vs. 5.0	0.48 (0.36–0.64)	NR vs. NR	0.61 (0.40–0.93)
Sintilimab + chemotherapy (*n* = 179)	Placebo + chemotherapy (*n* = 178)	ORIENT‐12 [43] (NCT03629925)	Squamous only	44.7 vs. 35.4	6.1 vs. 5.1	5.5 vs. 4.9	0.54 (0.42–0.68)	NR vs. NR	0.57 (0.35–0.91)
Sugemalimab+ chemotherapy (*n* = 320)	Placebo + chemotherapy (*n* = 159)	GEMSTONE 302 [44] (NCT03789604)	Squamous and nonsquamous	63.4 vs. 40.3	9.8 vs. 4.4	9.0 vs. 4.9	0.48 (0.39–0.60)	22.8 vs. 17.7	0.67 (0.50–0.90)
Tislelizumab + chemotherapy (*n* = 222)	Chemotherapy (*n* = 110)	RATIONALE 304 [45] (NCT03663205)	Nonsquamous	57.4 vs. 36.9	8.5 vs. 6.0	9.7 vs. 7.6	0.65 (0.46–0.90)	NR vs. NR	NE
Tislelizumab + chemotherapy (*n* = 120)	Chemotherapy (*n* = 121)	RATIONALE 307 [46] (NCT03594747)	Squamous	72.5 vs. 50.0	8.2 vs. 4.2	7.6 vs. 5.5	0.52 (0.37–0.74)	NR vs. NR	NE
Tremelimumab + durvalumab + chemotherapy (*n* = 338)	Chemotherapy (*n* = 337)	POSEIDON [47] (NCT03164616)	Squamous and nonsquamous	38.8 vs. 24.4	9.5 vs. 5.1	6.2 vs. 4.8	0.72 (0.60–0.86)	14.0 vs. 11.7	0.77 (0.65–0.92)
Nivolumab + ipilimumab (*n* = 396)^a^	Chemotherapy (*n* = 397)	CheckMate 227 [48, 49] (NCT02477826)	Squamous and nonsquamous	36.4 vs. 30.0	23.2 vs. 6.7	5.1 vs. 5.6	0.81 (0.68–0.96)	17.1 vs. 14.9	0.76 (0.65–0.90)
Nivolumab + ipilimumab + chemotherapy (*n* = 204)	Chemotherapy (*n* = 204)	CheckMate 9LA [50] (NCT03215706)	Squamous and nonsquamous	42.6 vs. 27.9	11.8 vs. 5.6	7.0 vs. 5.0	0.67 (0.53–0.84)	15.8 vs. 10.9	0.70 (0.56–0.89)

Abbreviations: CI, confidence interval; DOR, duration of response; HR, hazard ratio; ICI, immune checkpoint inhibitor; NA, not available; NCT, National Clinical Trial; NE, not evaluable; NR, not reached; NSCLC, non‐small cell lung cancer; ORR, objective response rate; OS, overall survival; PFS, progression‐free survival.

^a^ Study did not combine ICI with chemotherapy.

The multicenter Phase III KEYNOTE‐189 trial (NCT02578680) established the clinical rationale for integrating pembrolizumab with platinum‐pemetrexed chemotherapy in metastatic nonsquamous NSCLC [51]. Interim analysis demonstrated superior 12‐month survival probabilities (69.2% vs. 49.4%) and prolonged median PFS (8.8 vs. 4.9 months; HR = 0.52, 95% CI = 0.43–0.64) favoring the immunochemotherapeutic regimen. These practice‐changing outcomes underpinned the FDA's 2018 regulatory authorization (August 20; BLA 125514) for this combination as frontline therapy in nonsquamous NSCLC [52]. Subsequent 60‐month follow‐up data reinforced sustained survival advantages (5‐year OS: 19.4% vs. 11.3%), validating durable clinical benefit [53]. Given therapeutic limitations in squamous histology, the parallel KEYNOTE‐407 trial (NCT02775435) evaluated carboplatin‐paclitaxel combinations with pembrolizumab [30, 54]. This biomarker‐unselected population showed significant OS improvement (15.9 vs. 11.3 months; HR = 0.64, *p* < 0.001) and PFS extension (6.4 vs. 4.8 months; HR = 0.56, *p* < 0.001), leading to FDA approval (October 30, 2018; STN 125514/Orig1s018) for squamous NSCLC [55]. Current NCCN guidelines designate pembrolizumab‐based immunochemotherapy as the exclusive PD‐1 inhibitor regimen recommended for squamous and nonsquamous histology as first‐line management [12].

The PD‐L1 inhibitor atezolizumab has secured regulatory authorization as monotherapy for first‐line NSCLC management, with accumulating evidence supporting its combinatorial potential. The Phase III IMpower130 trial (NCT02367781) established significant clinical benefit for atezolizumab combined with carboplatin/nab‐paclitaxel vs. chemotherapy alone in nonsquamous NSCLC, demonstrating a notable enhancement in OS, reporting 18.6 months compared to 13.9 months, as well as an extension in PFS from 5.5 to 7.0 months, alongside a higher response rate, which increased from 31.9% to 49.2%, when comparing first‐line atezolizumab plus chemotherapy to chemotherapy alone [31]. Building upon these findings, the IMpower150 trial (NCT02366143) evaluated a novel quadruplet regimen (atezolizumab+bevacizumab+carboplatin+paclitaxel) vs. standard bevacizumab‐chemotherapy in EGFR/ALK wild‐type nonsquamous NSCLC [32]. Although initial OS analysis showed non‐significant improvement (19.8 vs. 14.9 months; HR = 0.84, *p* = 0.06), extended 39.8‐month follow‐up revealed persistent survival curves separation (median OS 19.5 vs. 14.7 months). These collective data supported FDA accelerated approval (December 6, 2018; BLA 761034Orig1s014) for the four‐drug combination in PD‐L1‐positive (SP142 assay ≥ 1% TC/IC) metastatic nonsquamous NSCLC [56].

Cemiplimab, an inhibitor of PD‐1, demonstrated significant effectiveness both as a standalone treatment and combination therapy in the EMPOWER‐Lung 3 trial [36]. This Phase 3 clinical investigation compared the outcomes of cemiplimab in combination with platinum‐doublet chemotherapy against chemotherapy alone in individuals diagnosed with advanced nonsquamous or squamous NSCLC. The combination therapy of cemiplimab resulted in markedly improved OS rates, showing 21.9 months compared to 13.0 months, which corresponds to a 29% reduction in the risk of mortality. Additionally, there was an increase in the median PFS duration, recorded at 8.2 months vs. 5.0 months, alongside a longer duration of response (DOR) of 15.6 months compared to 7.3 months. The objective response rate (ORR) was also enhanced, with figures of 43.3% for the combination group as opposed to 22.7% for chemotherapy alone. This study was prematurely terminated because of the markedly improved survival outcomes associated with cemiplimab. The findings from this investigation subsequently resulted in an extended FDA approval for cemiplimab, allowing its use in combination with platinum‐based chemotherapy as first‐line treatment for patients with advanced NSCLC lacking EGFR, ALK, or ROS1 mutations as of November 2022 [57].

China‐developed ICIs have made contributions tailored to NSCLC treatment, and numerous combination regimens involving domestic ICIs have been investigated in China, which has provided more treatment options for both regional and global therapeutic needs. The therapeutic efficacy and safety profile of the novel PD‐1 inhibitor camrelizumab, when used alongside chemotherapy, was assessed in advanced NSCLC of both nonsquamous and squamous histologies through two randomized Phase 3 clinical trials, termed CameL and CameL‐sq, respectively [40, 41]. The findings from these studies indicated that the integration of camrelizumab with chemotherapy resulted in a statistically significant enhancement in both median PFS and OS compared to chemotherapy alone (Table 2). Notably, Grade ≥ 3 TRAEs were observed more frequently in patients with nonsquamous NSCLC receiving the camrelizumab combination (69% vs. 47%) compared to those treated with chemotherapy alone, while for squamous NSCLC, the rates were similar (74% vs. 72%). Furthermore, the anti‐PD‐1 antibody tislelizumab was also evaluated in conjunction with chemotherapy in advanced NSCLC patients in China across two separate randomized Phase 3 trials [45, 46]. The RATIONALE 304 trial focused on nonsquamous NSCLC, while RATIONALE 307 concentrated on squamous NSCLC. The results demonstrated that tislelizumab with chemotherapy yielded significantly improved median PFS, a higher ORR, and an extended DOR when juxtaposed with chemotherapy alone in both studies, while showing a manageable safety profile. In the Phase 3 ORIENT‐11 trial, another PD‐1 inhibitor, sintilimab, was investigated in combination with chemotherapy vs. chemotherapy alone as a first‐line treatment for Chinese patients diagnosed with locally advanced or metastatic nonsquamous NSCLC [42]. The patients receiving the sintilimab combination exhibited a longer PFS (8.9 months vs. 5.0 months) and a higher ORR (51.9% vs. 29.8%) compared to their chemotherapy‐only counterparts. In the ORIENT‐12 trial involving squamous NSCLC, comparable outcomes were observed. The combination of sintilimab and chemotherapy resulted in an increased PFS of 5.5 months in contrast to 4.9 months for chemotherapy alone [43]. The CHOICE‐01 trial demonstrated that toripalimab, an anti‐PD‐1 monoclonal antibody, exhibited favorable results when paired with chemotherapy. Specifically, the toripalimab and chemotherapy combination yielded a median PFS of 8.4 months compared to 5.6 months for chemotherapy alone, along with an OS that was not yet reached vs. 17.1 months for the control group [34]. In Phase 3 ASTRUM‐004 trial, serplulimab, another anti‐PD‐1 antibody, demonstrated efficacy when administered alongside carboplatin and albumin‐bound paclitaxel [38, 39]. Additionally, penpulimab, a different PD‐1 inhibitor, showed efficacy in patients with advanced squamous NSCLC in the Phase 3 AK105‐302 trial [35]. The penpulimab and carboplatin‐paclitaxel regimen resulted in a median PFS of 7.6 months, as opposed to 4.2 months for chemotherapy alone, along with improved 12‐month (37.1% vs. 9.2%) and 24‐month PFS rates (23.8% vs. 5.9%).

Beyond these anti‐PD‐1 agents, sugemalimab, an anti‐PD‐L1 antibody, was investigated in first‐line treatment settings in conjunction with chemotherapy vs. chemotherapy alone in metastatic NSCLC within the GEMSTONE‐302 trial [44]. The combination therapy with sugemalimab showed a longer median PFS than chemotherapy alone for both squamous (8.3 vs. 4.8 months) and nonsquamous (9.6 vs. 5.8 months) NSCLC patients, independent of PD‐L1 expression levels. Specifically, the median PFS for patients with PD‐L1 expression levels of < 1%, ≥ 1%, 1%–49%, and ≥ 50% were recorded at 7.4, 10.9, 8.8, and 12.9 months, respectively, when treated with sugemalimab combination therapy. The safety profile appeared to be comparable between the groups receiving sugemalimab in conjunction with chemotherapy and those receiving chemotherapy alone.

Meanwhile, domestic innovative multi‐target agents, such as ivonescimab, a bispecific antibody that targets PD‐1 and VEGF, have demonstrated clinical efficacy comparable to pembrolizumab in advanced NSCLC with positive PD‐L1 expression (PD‐L1 TPS ≥ 1%) in the first‐line setting [58]. Also, the clinical study of cadonilimab (a domestic PD‐1/CTLA‐4 bispecific antibody) in treating treatment‐naïve advanced NSCLC (CTR20232128) is actively recruiting, which shows a new paradigm in NSCLC immunotherapy that balances efficacy, safety, and accessibility and provides critical insights for global precision immunotherapy.

Drawing from the clinical investigations, camrelizumab, tislelizumab, sintilimab, sugemalimab, toripalimab, serplulimab, and penpulimab, when administered alongside chemotherapy, have received approval in China for the management of advanced NSCLC of both nonsquamous and squamous types [42, 43, 45, 46, 59, 60, 61, 62, 63, 64, 65]. These developments are supported by domestic robust translational research infrastructure and offer the broader array of therapeutic options for patients with advanced NSCLC. However, challenges remain in comparing the effectiveness and managing adverse events in genetically distinct populations from large‐scale real‐world studies in China.

### 3.3 Clinical Trials of First‐Line Immunotherapy Combination Therapy Without Chemotherapy

PD‐1 and CTLA‐4 each contribute to distinct yet complementary immunological pathways, prompting significant interest in their combined utilization for cancer therapy [48, 66]. The integration of these immunotherapeutic strategies has demonstrated enhanced efficacy in NSCLC relative to monotherapy with ICIs [10]. Notably, nivolumab, an anti‐PD‐1 monoclonal antibody, with ipilimumab, an anti‐CTLA‐4 monoclonal antibody, has shown substantial improvements and is recommended as a first‐line treatment for patients with advanced NSCLC (Table 2) [67, 68].

The CheckMate 227 trial, part 1, assessed the efficacy and safety of nivolumab plus ipilimumab in comparison to nivolumab monotherapy or chemotherapy in patients diagnosed with treatment‐naïve advanced NSCLC [48]. The combination therapy of nivolumab and ipilimumab yielded a statistically significant extension in median OS, recorded at 17.1 months compared to 14.9 months for chemotherapy. Additionally, the two‐year OS rate was notably higher in the combination group (40.0%) as opposed to the chemotherapy‐only group (32.8%). Furthermore, the median DOR was substantially longer in the nivolumab plus ipilimumab cohort, at 23.2 months, in contrast to 6.2 months observed with chemotherapy. It was also noted that the incidence of Grade 3/4 TRAEs was lower in the nivolumab plus ipilimumab group, accounting for 32.8% compared to 36.0% in the chemotherapy group.

In the Phase 3 CheckMate 9LA trial, the combination of nivolumab and ipilimumab along with two cycles of chemotherapy demonstrated a significantly prolonged median OS compared to chemotherapy alone during the interim analysis (median follow‐up of 9.7 months; median OS of 14.1 vs. 10.7 months). This was further confirmed in the subsequent analysis (median follow‐up of 30.7 months; median OS of 15.8 vs. 11.0 months) [50, 69]. As a result, this combination therapy received FDA approval in May 2020 [70]. Despite the observed efficacy of nivolumab plus ipilimumab, with or without chemotherapy, it is noteworthy that the incidence of Grade 3/4 adverse AEs was higher compared to those receiving ICI monotherapies [71].

The Phase 3 POSEIDON trial evaluated the efficacy of first‐line treatment comprising durvalumab, tremelimumab, and chemotherapy in comparison to chemotherapy alone in individuals diagnosed with metastatic NSCLC [47]. The combination of ICIs was associated with a notably longer median PFS of 6.2 months, compared to 4.8 months for chemotherapy alone, as well as an increase in median OS of 14.0 months vs. 11.7 months for the chemotherapy group. Grade 3 or 4 TRAEs were reported in 51.8% of patients receiving the ICI combination therapy, in contrast to 44.4% in those treated with chemotherapy only. Considering these findings, the US FDA granted approval for the use of tremelimumab in conjunction with durvalumab and platinum‐based chemotherapy for patients with metastatic NSCLC in November 2022 [72].

## 4 Clinical Trials of Other Novel Targets

It is widely recognized that PD‐1 and PD‐L1 are critical in modulating T‐cell activity. Although ICIs have resulted in substantial enhancements in patient survival rates, a significant number of individuals were without long‐lasting responses, and their conditions demonstrate resistance to anti‐PD‐(L)1 treatment [73]. Furthermore, various interactions between co‐inhibitory receptor‐ligand pairs can impede the antitumor efficacy of CD8^+^ T cells, affecting their functions both directly and indirectly. Notable co‐inhibitory receptors in this context include the T‐cell immunoreceptor with Ig and ITIM domains (TIGIT), lymphocyte activation gene 3 protein (LAG‐3), and CTLA‐4, which represent promising avenues for immunotherapy in NSCLC (Figure 1B).

### 4.1 TIGIT‐Targeted Therapy

TIGIT is notably present in various lymphocyte populations, including CD8^+^ T cells, both memory and regulatory CD4^+^ T cells, follicular CD4+ T cells, and natural killer cells [73]. CD155, the primary ligand for TIGIT, is found on tumor‐infiltrating myeloid cells and is upregulated on tumor cells, which facilitates local immune surveillance suppression. Although targeting TIGIT alone has demonstrated limited effectiveness in clinical trials, the combination of TIGIT inhibition with PD‐(L)1 blockade has shown enhanced efficacy, functioning as an immune amplifier to counteract immune suppression. A few TIGIT‐targeting agents and combination therapies have been evaluated in the first‐line treatment of advanced NSCLC.

Tiragolumab, a TIGIT antagonist, was assessed in conjunction with atezolizumab within the CITYSCAPE and SKYSCRAPER‐01 clinical trials. The Phase 2 CITYSCAPE study yielded encouraging outcomes, demonstrating enhancements in PFS and ORR when comparing the tiragolumab‐atezolizumab combination to atezolizumab monotherapy; nevertheless, larger‐scale investigations are necessary for validation [74]. During the interim analysis of the Phase 3 SKYSCRAPER‐01 trial, the co‐primary endpoint of PFS was not achieved, and the other co‐primary endpoint of OS was considered immature [75]. Despite this, both co‐primary endpoints exhibited numerical advantages, and additional analyses are currently in progress.

Vibostolimab, an additional TIGIT inhibitor, has been investigated in conjunction with pembrolizumab within the framework of the KeyVibe‐007 and KeyVibe‐003 clinical trials. The Phase 3 KeyVibe‐007 trial aimed to compare the efficacy and safety profiles of vibostolimab combined with pembrolizumab and chemotherapy against those of pembrolizumab paired with chemotherapy alone [76]. In a parallel Phase 3 trial, KeyVibe‐003 is currently underway, focusing on PFS and OS as co‐primary end points, while also evaluating ORR, DOR, and safety as secondary endpoints [77]. The findings from these investigations are currently awaited and have yet to be disclosed or published.

AdvanTIG‐105 represents a Phase 1b dose‐expansion trial aimed at assessing the efficacy and safety of ociperlimab, which is another TIGIT inhibitor, in conjunction with tislelizumab [78]. This combination exhibited a satisfactory safety profile, as most of the TRAEs were classified as Grade 1 or 2, alongside demonstrating antitumor activity in the first‐line treatment context. While this study is in its preliminary phase and involves a limited number of participants, the results obtained indicate a level of promise that justifies additional research.

ARC‐7 represents a Phase 2 clinical trial that investigates the effectiveness and safety of domvanalimab, a TIGIT inhibitor, when used in conjunction with zimberelimab, a PD‐1 inhibitor [79]. The combination of domvanalimab demonstrated a manageable safety profile and yielded significant enhancements in ORR and PFS when compared to zimberelimab administered alone (ORR: 41% vs. 27%; median PFS: 12.0 months vs. 5.4 months). Ongoing Phase 3 trials are currently assessing the outcomes associated with this combination treatment strategy.

### 4.2 Bispecific Antibody Targeted Therapy

The exploration of combined PD‐(L)1 and CTLA‐4 antagonists is currently emerging as a promising avenue for bispecific therapies. Cadonilimab (AK104), which functions as a PD‐1/CTLA‐4 bispecific antibody, is under investigation in a Phase 1b/2 clinical trial in conjunction with anlotinib for patients with advanced NSCLC [80]. The findings indicated a promising safety profile alongside an ORR of 70.6% in treatment‐naive individuals. Similarly, MEDI5752, another bispecific antibody targeting PD‐1 and CTLA‐4, was evaluated in a Phase 1b/2 trial, yielding superior DOR, PFS, and OS compared to pembrolizumab [81]. However, a notable incidence of Grade 3 TRAEs (70%) prompted investigations into a reduced dosage of MEDI5752, which demonstrated enhanced tolerability while maintaining comparable efficacy. Additionally, KN046, another bispecific antibody targeting PD‐L1 and CTLA‐4, exhibited encouraging clinical outcomes in Phase 2 trials when administered in combination with chemotherapy, particularly among tumors with PD‐L1 expression ≥ 1% and squamous histology [82].

Research into bispecific antibodies targeting PD‐(L)1 alongside additional targets is actively ongoing. Ivonescimab (AK112), a bispecific antibody that targets PD‐1 and VEGF, has been assessed as a first or second‐line treatment for advanced NSCLC, with preliminary findings indicating notable antitumor efficacy and a manageable safety profile [83]. In November 2022, AK112 received breakthrough therapy designation in conjunction with docetaxel for patients in China suffering from advanced NSCLC and exhibiting resistance to PD‐(L)1 immunotherapy [84]. The Phase 3 clinical trial (HARMONi‐2 or AK112‐303) was designed to compare the efficacy of AK112 monotherapy against pembrolizumab as a first‐line treatment in cases of locally advanced or metastatic NSCLC with positive PD‐L1 expression (PD‐L1 TPS ≥ 1%). Preliminary results, as reported by an independent data monitoring committee (IDMC), have shown encouraging outcomes [58]. The primary endpoint of the study, PFS, was met with significant efficacy, revealing a median PFS of 11.14 months compared to 5.82 months (HR = 0.51, *p* < 0.0001). Furthermore, PF‐07257876 and IBI322, both PD‐L1/CD47 bispecific antibodies, are presently under investigation in distinct Phase 1 trials (NCT04881045 and NCT04795128, respectively) to assess their safety and tolerability across various cancer types. Initial findings suggest that IBI322 is well tolerated, with 33% of the nine NSCLC patients receiving this treatment achieving a partial response [85]. The findings from these studies involving novel bispecific agents may pave the way for innovative approaches in ICIs for the management of advanced NSCLC.

### 4.3 LAG‐3‐Targeted Therapy

LAG‐3 represents another promising candidate for ICIs, as it is expressed on the surfaces of both effector and regulatory T cells, which are vital components of the adaptive immune response [86]. The interaction between LAG‐3 and additional inhibitory receptors, such as PD‐1 and CTLA‐4, may improve the immunosuppressive functions of regulatory T cells, ultimately facilitating immune tolerance mediated by antigen‐presenting cells. Inhibition of LAG‐3 has been shown to restore the cytotoxic capabilities of T cells while reducing the suppressive effects exerted by regulatory T cells, thereby increasing tumor‐specific cytotoxicity. Furthermore, elevated expression levels of LAG‐3 have been correlated with unfavorable prognostic outcomes in NSCLC [87]. Several therapies targeting LAG‐3, including favezelimab, eftilagimod alpha, and relatlimab, are currently being assessed in recent clinical trials for first‐line treatment in advanced NSCLC.

Favezelimab was evaluated in conjunction with pembrolizumab during the Phase 2 KEYNOTE‐495 trial [88]. According to the interim analysis, the confirmed ORR was recorded at 23% for the 200 mg dosage of favezelimab, while the 800 mg dosage yielded an ORR of 26%. Notably, the highest ORR for the 200 mg dose reached 60% among patients exhibiting a high T‐cell‐inflamed gene expression profile alongside a high tumor mutational burden (TMB). Conversely, the 800 mg dose demonstrated a peak ORR of 50% in patients characterized by a low T‐cell‐inflamed gene expression profile yet possessing a high TMB. Consequently, further extensive investigations are necessary to evaluate the therapeutic outcomes associated with favezelimab in NSCLC.

TACTI‐002 is a Phase 2 clinical trial that explores the efficacy of eftilagimod alpha in conjunction with pembrolizumab as a first‐line treatment for metastatic NSCLC [89]. Preliminary findings demonstrated promising antitumor effects, evidenced by an ORR of 37.3% within the intention‐to‐treat cohort and a notably higher ORR of 54.5% among patients exhibiting PD‐L1 expression levels of 50% or greater. These results justify the need for additional research into this combinatorial therapeutic approach.

The efficacy and safety profile of the first‐line combination of relatlimab, nivolumab, and chemotherapy is currently under investigation and is being compared to that of nivolumab combined with chemotherapy in previously untreated patients with advanced NSCLC in the Phase 2 RELATIVITY‐104 trial [90]. As this study is still in progress, results have yet to be disclosed.

## 5 Biomarkers for Predicting Efficacy

### 5.1 Treatment Options in Patients With Actionable Mutation Status

Once the histological subtype has been verified, the subsequent focus shifts to the evaluation of therapy‐predictive biomarkers [11]. It is advisable to conduct molecular testing for mutations and alterations involving EGFR, ALK, KRAS, ROS1, BRAF, NTKR, and METex14 skipping, within the framework of comprehensive molecular profiling [11, 12, 91]. In general, patients presenting with targetable mutations or alterations are typically recommended to receive targeted therapies as their primary treatment approach.

### 5.2 Treatment Options in Patients With Different PD‐L1 Expression Levels

**FIGURE 2:**
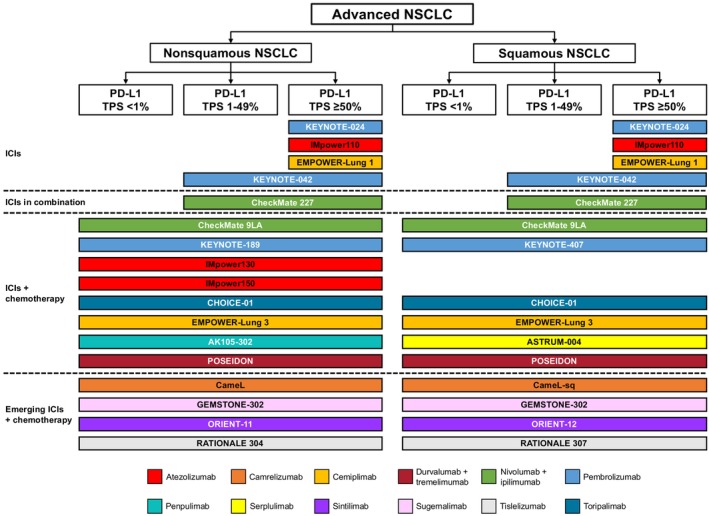
First‐line ICI‐based treatment landscape for patients with advanced NSCLC. Diagram shows the ICI investigated in each clinical trial and the histological subtype and PD‐L1 expression level(s) of the patients included. ICI, immune checkpoint inhibitor; NSCLC, non‐small cell lung cancer; PD‐L1, programmed cell death 1 ligand 1; TPS, tumor proportion score.

Incorporating the assessment of PD‐L1 expression levels, as determined through immunohistochemical techniques, is essential in formulating treatment strategies for patients considering ICIs. A substantial body of evidence indicates that stratifying patients based on PD‐L1 expression, in conjunction with histological characteristics and the severity of the disease, plays a crucial role in guiding the selection of initial therapeutic interventions. Figure 2 provides a comprehensive overview of Phase 3 clinical trials that evaluate various ICIs administered as first‐line treatments, either as monotherapies or in combination therapies, targeting patients with advanced NSCLC among different PD‐L1 expression levels.

Around 30% of individuals diagnosed with advanced NSCLC who do not exhibit EGFR or ALK genomic alterations demonstrate a PD‐L1 expression level exceeding 50% [48, 69, 92]. In patients presenting with PD‐L1 ≥ 50% or higher and lacking any actionable oncogenic drivers, the utilization of single‐agent therapies has yielded superior treatment outcomes in comparison to conventional chemotherapy (Table 3) [93]. The approval of pembrolizumab was predicated upon findings from the KEYNOTE‐024 trial, which indicated enhanced efficacy outcomes when pembrolizumab was administered as opposed to chemotherapy alone in patients with PD‐L1 expression ≥ 50% [17, 94]. Similarly, both atezolizumab and cemiplimab have received approval for first‐line treatment in patients exhibiting PD‐L1 levels of 50% or greater, with no evidence of EGFR or ALK alterations, based on the outcomes from the Phase 3 IMpower110 trial and the Phase 3 EMPOWER‐Lung 1 trial, respectively [21, 25, 26, 27, 95].

**TABLE 3:** Efficacy of first‐line treatment options in patients with advanced NSCLC based on PD‐L1 status.

Drug	Comparator	Study (NCT #)	ORR, %	DOR, median, months	PFS		OS	
					Median, months	HR (95% CI)	Median, months	HR (95% CI)
PD‐L1 ≥ 50%								
Atezolizumab (*n* = 107)	Chemotherapy (*n* = 98)	IMpower110 [20] (NCT02409342)	38.3 vs. 28.6	NE	8.1 vs. 5.0	0.63 (0.45–0.88)	20.2 vs. 13.1	0.59 (0.40–0.89)
Cemiplimab (*n* = 283)	Chemotherapy (*n* = 280)	EMPOWER‐Lung 1 [21] (NCT03088540)	39 vs. 20	16.7 vs. 6.0	8.2 vs. 5.7	0.54 (0.43–0.68)	NR vs. 14.2	0.57 (0.42–0.77)
Pembrolizumab (*n* = 154)	Chemotherapy (*n* = 151)	KEYNOTE‐024 [17, 18] (NCT02142738)	44.8 vs. 27.8	NR vs. 6.3	10.3 vs. 6.0	0.50 (0.37–0.68)	30.0 vs. 14.2	0.63 (0.47–0.86)
Pembrolizumab (*n* = 299)	Chemotherapy (*n* = 300)	KEYNOTE‐042 [19] (NCT02220894)	39 vs. 32	20.2 vs. 10.8	7.1 vs. 6.4	0.81 (0.67–0.99)	20.0 vs. 12.2	0.69 (0.56–0.85)
PD‐L1 ≥ 1%								
Pembrolizumab (*n* = 637)	Chemotherapy (*n* = 637)	KEYNOTE‐042 [19] (NCT02220894)	27 vs. 27	20.2 vs. 8.3	5.4 vs. 6.5	1.07 (0.94–1.21)	16.7 vs. 12.1	0.81 (0.71–0.93)
Nivolumab + ipilimumab (*n* = 396)	Chemotherapy (*n* = 397)	CheckMate 227 [48, 49] (NCT02477826)	36.4 vs. 30.0	23.2 vs. 6.7	5.1 vs. 5.6	0.81 (0.68–0.96)	17.1 vs. 14.9	0.76 (0.65–0.90)
Nivolumab + ipilimumab + chemotherapy (*n* = 204)	Chemotherapy (*n* = 204)	CheckMate 9LA [50, 69] (NCT03215706)	42.6 vs. 27.9	11.8 vs. 5.6	7.0 vs. 5.0	0.67 (0.53–0.84)	15.8 vs. 10.9	0.70 (0.56–0.89)

Abbreviations: CI, confidence interval; DOR, duration of response; HR, hazard ratio; NCT, National Clinical Trial; NE, not evaluable; NR, not reached; NSCLC, non‐small cell lung cancer; ORR, objective response rate; OS, overall survival; PD‐L1, programmed cell death ligand 1; PFS, progression‐free survival.

The suggested therapeutic strategies for individuals presenting with PD‐L1 levels below 50% encompass various combination immunotherapy regimens, which may involve an alternative ICI or chemotherapy, alongside the option of pembrolizumab as a standalone treatment. The indication for pembrolizumab as a monotherapy was broadened for patients exhibiting PD‐L1 expression ≥ 1% or higher, predicated on the efficacy advantage demonstrated in the Phase 3 KEYNOTE‐042 trial when compared to chemotherapy [19, 24]. Following this, subsequent Phase 3 trials have illustrated the efficacy of ICI combination treatments in patients with advanced NSCLC, regardless of PD‐L1 expression levels [29, 30, 31, 32, 40, 48, 49, 50, 69].

### 5.3 Treatment Options in Patients With Different TMB Statuses

TMB represents a crucial biomarker utilized for forecasting the efficacy of ICI therapy in individuals diagnosed with NSCLC [96]. Prior investigations have indicated a positive correlation between elevated TMB levels and enhanced ORR, as well as extended PFS and OS in patients receiving ICI treatment. Conversely, some research suggests that high TMB may be linked to poorer clinical outcomes. To elucidate the predictive capability of TMB as an efficacy biomarker for ICI therapy in NSCLC patients, a meta‐analysis was conducted. The findings of this study revealed that NSCLC patients exhibiting elevated TMB levels experience substantial clinical advantages from ICI therapy compared to those with lower TMB. Additionally, another meta‐analysis yielded comparable results, demonstrating that patients with heightened TMB experienced increased PFS and OS, alongside improved ORR compared to their lower TMB counterparts [97]. Such analyses highlight the association between higher TMB and improved survival outcomes in patients undergoing ICI treatment; nevertheless, further research is warranted to solidify these findings.

### 5.4 Impact of Microbiome, Diet, and Antibiotic Use on ICI Efficacy

Emerging evidence highlights the critical interplay between gut microbiota, dietary patterns, and antibiotic exposure in responses to ICIs. Recent studies suggest that these factors may influence treatment outcomes by modulating both systemic immunity and the tumor microenvironment, offering new avenues for personalized immunotherapy strategies.

The gut microbiome has emerged as a key predictor of ICI efficacy [98, 99]. One small cohort study focused on NSCLC and suggests that gut microbiota metabolic pathways could affect the response to immunotherapy for NSCLC patients [100]. Diet and antibiotic drugs can influence the abundance of gut microbiota and therefore influence the efficacy of ICI treatment in NSCLC [101]. Some studies suggested that antibiotic use is an independent predictor of shorter PFS and OS in patients with advanced cancer treated with ICIs [102, 103, 104]. Targeted modification of the gut microbiota and dietary intervention could become an innovative strategy for enhancing cancer immunotherapy efficacy [105], and fecal microbiota transplantation as an adjunct therapy in immunotherapy is explored [106].

## 6 Concerns With Choosing ICI Treatment

### 6.1 Evidence Gap in Efficacy Complicating ICI Sequencing

Optimal sequencing of immune checkpoint blockade strategies in advanced NSCLC constitutes complex clinical decision‐making, predominantly constrained by the lack of head‐to‐head clinical trials evaluating comparative effectiveness between monotherapeutic and combinatorial ICI approaches [107]. A network meta‐analysis encompassing 22 randomized controlled trials conducted indirect efficacy comparisons between ICI monotherapies and ICIs combined with chemotherapy specifically for NSCLC patients exhibiting high levels of PD‐L1 expression [108]. The findings from this analysis indicated that the combination of ICI and chemotherapy was linked to a significantly enhanced ORR and PFS when juxtaposed with ICI monotherapy; however, no substantial difference in OS was observed.

### 6.2 Dilemmas in Balancing Efficacy and Safety

When making the treatment decision in first‐line therapy, not only the efficacy, but also the safety of ICI treatment should be addressed. The incidence of Grade 3/4 AEs associated with ICI combination therapies was generally elevated in comparison to ICI monotherapies (Table 4) [17, 18, 19, 29, 48, 49, 50, 69]. A meta‐analysis suggested that in NSCLC patients, PD‐L1 positivity may predict increased risks of Grade 3 and 4 TRAEs and AEs of ICI treatment [109]. For the association of ICI treatment and TRAEs, another meta‐analysis suggested that no matter ICI monotherapies or combination therapies, compared with placebo/best supportive care, PD‐1/PD‐L1 inhibitors were significantly correlated with a higher possibility of TRAEs [110]. These studies indicated the importance of pre‐treatment evaluation of patients and personalized the treatment plan for ICI treatment.

**TABLE 4:** Safety for first‐line ICI therapies in patients with advanced NSCLC.

Drug	Study (NCT #)	Safety population, *n*	Any‐grade TRAEs, %	Grade 3/4 TRAEs, %
Atezolizumab	IMpower110 [20] (NCT02409342)	286	90.2^a^	30.1^a^
Atezolizumab + chemotherapy	Impower130 [31] (NCT02367781)	473	96.2	73.2
Atezolizumab + chemotherapy	Impower150 [32] (NCT02366143)	400	94.3	43.0
Camrelizumab + chemotherapy	CameL [40] (NCT03134872)	205	< 100.0	68.8 (Grades 3–5)
Camrelizumab + chemotherapy	CameL‐sq [41] (NCT03668496)	193	100.0	73.6
Cemiplimab	EMPOWER‐Lung 1 [21] (NCT03088540)	355	57.5	11.5
Cemiplimab + chemotherapy	EMPOWER‐Lung 3 [36, 37] (NCT03409614)	312	96.5	48.7 (Grades 3–5)
Durvalumab + tremelimumab + chemotherapy	POSEIDON [47] (NCT03164616)	330	92.7	51.8
Nivolumab + ipilimumab	CheckMate 227 [48, 49] (NCT02477826)	576	76.7	32.8
Nivolumab + ipilimumab + chemotherapy	CheckMate 9LA [50, 69] (NCT03215706)	358	91.6	48.3
Pembrolizumab	KEYNOTE‐024 [17, 18] (NCT02142738)	154	73.4	26.6 (Grades 3–5)
Pembrolizumab	KEYNOTE‐042 [19] (NCT02220894)	636	62.7	17.8 (Grades 3–5)
Pembrolizumab + chemotherapy	KEYNOTE‐189 [29, 51] (NCT02578680)	405	99.8^a^	71.9^a^ (Grades 3–5)
Pembrolizumab + chemotherapy	KEYNOTE‐407 [30, 54] (NCT02775435)	278	98.6^a^	56.5 (Grades 3–5)
Penpulimab + chemotherapy	AK105‐302 [35] (NCT03866993)	175	NA	63.6 (Grades 3–5)
Serplulimab + chemotherapy	ASTRUM‐004 [38, 39] (NCT04033354)	NA	NA	NA
Sintilimab + chemotherapy	ORIENT‐11 [42] (NCT03607539)	266	99.6^a^	61.7^a^ (Grades 3–5)
Sintilimab + chemotherapy	ORIENT‐12 [43] (NCT03629925)	179	100.0^a^	86.6^a^ (Grades 3–5)
Sugemalimab + chemotherapy	GEMSTONE 302 [44] (NCT03789604)	320	99.1	53.8
Tislelizumab + chemotherapy	RATIONALE 304 [45] (NCT03663205)	222	100.0^a^	67.6^a^ (Grades 3–5)
Tislelizumab + chemotherapy	RATIONALE 307 [46] (NCT03594747)	120	99.2	85.8
Toripalimab + chemotherapy	CHOICE‐01 [34] (NCT03856411)	308	99.0^a^	78.6^a^ (Grades 3–5)

Abbreviations: ICI, immune checkpoint inhibitor; NA, not available; NCT, National Clinical Trial; NSCLC, non‐small cell lung cancer; TRAE, treatment‐related adverse event.

^a^ May include adverse events that are not treatment related.

Although previous studies indicated that multisystem irAEs were associated with improved survival in patients with advanced NSCLC receiving ICI treatment, serious (Grades 3 and 4) AEs could cause the discontinuation or termination of treatment and have an impact on survival [111, 112]. Key challenges and future prospects in toxicity management include exploring reliable biomarkers for irAE risk stratification and balancing irAEs and ICI efficacy. Biomarkers for irAEs vary, including circulating blood cell counts, cytokines, autoantibodies, serum proteins, HLA genotypes, microRNA and gene profiling, and intestinal microbiota [113]. These explorations of irAE biomarkers might provide some clues for preventing irAEs. For managing irAEs, corticosteroids remain the standard of care therapy [114]. However, high doses and long‐duration usage of corticosteroids could hamper the efficacy of ICI and cause other unexpected adverse events. With the exploration of the mechanism of irAEs, some new treatment strategies, such as TNF blockade, interleukin‐23 blockade, and anti‐interleukin‐6 receptor that could attenuate immunotoxicity and preserve antitumor immunity were undergoing clinical trials [115, 116, 117]. These ongoing studies revealed unexplored parts of ICI therapy and could provide more insight into balancing safety and efficacy in clinical practice when making treatment decisions.

### 6.3 Treatment Selection Barriers in Specific Populations

Since the initial approval of nivolumab as an ICI by the FDA for the treatment of advanced NSCLC [118], the introduction of novel ICI therapies has markedly enhanced patient outcomes. Nevertheless, the median OS observed in Phase 3 clinical studies typically falls between 14.0 and 30.0 months, suggesting a potential plateau in survival benefits. Additionally, factors such as patient age and performance status have a significant impact on treatment selection and, consequently, on survival rates. Data from the SEER database indicate that 47% of NSCLC patients are aged over 70, with 14% exceeding 80 years of age [119]. In older individuals, the presence of poor Eastern Cooperative Oncology Group performance status and various complex comorbidities may render the combination of ICI and chemotherapy an impractical treatment option. Furthermore, the exclusion of elderly patients from many clinical trials has resulted in a deficit of clinical evidence regarding the application of ICIs in this demographic. For first‐line treatment in elderly patients, several studies have assessed the efficacy and safety of ICI combination therapy in comparison to ICI monotherapy and single‐agent chemotherapy [20, 31, 51, 54, 120]. In a real‐world investigation conducted in Japan, known as NEJ057, the median OS was reported to be 20.0 months for the ICI plus chemotherapy cohort, 19.8 months for the ICI monotherapy group, 12.8 months for those receiving platinum‐doublet chemotherapy, and 9.5 months for patients treated with single‐agent chemotherapy [121]. The findings from this study suggest that ICI monotherapy may exhibit similar efficacy to that of ICI combination therapy, highlighting the necessity for ongoing research to innovate new therapeutic strategies, refine the timing of ICI combination regimens, and assess adverse effects through extensive clinical trials and real‐world investigations.

### 6.4 Deficiencies in Biomarker Utility Complicating Drug Selection

Furthermore, it is essential to investigate the selection between anti‐PD‐1 and anti‐PD‐L1 therapies. While existing evidence indicates that both drug classes demonstrate efficacy in first‐line treatment regimens, a meta‐analysis revealed that anti‐PD‐1 agents are correlated with improved overall survival (OS) in comparison to anti‐PD‐L1 agents. This finding underscores the necessity for head‐to‐head comparisons to further elucidate their relative effectiveness [122].

Even though PD‐L1 expression currently serves as the most reliable biomarker for informing treatment choices, it lacks a direct association with specific patient subsets or distinct disease traits. A comprehensive review of the literature revealed no significant correlation between PD‐L1 expression and variables such as gender, age, smoking history, tumor histology, performance status, tumor grade, or mutation status [123]. Furthermore, the methodologies employed to assess PD‐L1 expression in advanced NSCLC tissues exhibit considerable variability across different clinical trials. Future investigations must be adequately powered to include both PD‐L1‐positive and ‐negative patients, while also employing standardized and validated assays for the evaluation of PD‐L1 expression. Although numerous studies have indicated a relationship between elevated PD‐L1 expression and unfavorable survival outcomes, it is imperative that PD‐L1 is utilized solely as a biomarker for guiding treatment decisions, rather than as a prognostic indicator. The application of molecular testing for therapeutic selection remains underutilized in clinical practice, thereby decreasing the probability that healthcare providers will identify the most appropriate treatment options for their patients [124].

### 6.5 Socioeconomic Variables Confounding Treatment Access

In addition to the constraints posed by limited comparative studies within clinical trials and the complexities involved in identifying optimal ICI treatment protocols, socioeconomic determinants significantly influence the treatment decisions made by physicians. The approval status of ICIs is subject to considerable global variation; for instance, the National Medical Products Administration in China has sanctioned eight ICIs for the treatment of NSCLC, in contrast to the six ICIs approved by the US FDA [27, 70, 125, 126]. Moreover, the global landscape reveals discrepancies in drug availability and accessibility, which are heavily influenced by the differing healthcare systems in various regions [127]. The economic viability of a pharmaceutical agent is contingent upon multiple factors, including its associated costs, toxicity levels, and quality‐adjusted life years (QALYs); for a healthcare provider or insurance entity to endorse or reimburse a medication, it must satisfy specific financial criteria, which also fluctuate internationally. Countries such as Australia and the United Kingdom exemplify effective management of ICI costs while underscoring potential financial ramifications when evaluating combination therapies involving ICIs [128]. Additionally, a patient's socioeconomic background can significantly affect their treatment alternatives; for instance, in the United States, lung cancer patients lacking health insurance tend to experience poorer survival rates, a situation that can be partially attributed to restricted access to available treatment modalities [129].

## 7 Strategies After ICI Failure

Although first‐line ICIs improved the survival of NSCLC, still the majority of patients would experience disease progression due to primary or secondary resistance [130]. Common practice after ICI failure was chemotherapy, and the efficacy of conventional chemotherapy was unsatisfactory, with median OS ranging from 6.8 to 7.0 months [131, 132].

Treatment strategies were explored after ICI failure. Antiangiogenic agents, including nintedanib, ramucirumab, and anlotinib combined with chemotherapy, showed elevated median PFS and OS [133, 134, 135]. ICI‐rechallenge after first‐line ICI combined with chemotherapy was also explored, while the efficacy results were discouraging [136, 137, 138]. Novel targets and combination regimens were also explored, including antibody–drug conjugates (ADCs) drugs, bispecific antibodies, tumor‐infiltrating lymphocyte (TIL) therapy, and CAR‐T cell therapy. ADC drugs targeting trophoblast cell surface antigen 2 (TROP‐2), including datopotamab deruxtecan, sacituzumab govitecan, and SHR‐A1921, showed promising efficacy in post‐line treatment of advanced NSCLC and there are currently ongoing clinical trials exploring the efficacy of TROP‐2 ADC drugs for patients who failed first‐line chemo‐immunotherapy (NCT05687266, NCT05609968, and NCT06480136). Bispecific antibodies, including AK104 and KN046, mentioned before, have also shown efficacy in ICI‐failed patients and were undergoing clinical trials in ICI‐failed NSCLC patients (NCT03838848, NCT04995523, and NCT02517398). These therapeutic innovations targeting ICI‐failed NSCLC collectively bridge the translational gap between current clinical challenges and next‐generation solutions, offering not only salvage regimens for immediate application but also mechanistic blueprints for novel therapeutic discovery.

## 8 Conclusion

ICIs have emerged as a pivotal therapeutic advancement in the management of advanced NSCLC, significantly enhancing efficacy outcomes, including extended survival rates. Targets of ICIs, such as PD‐(L)1 and CTLA‐4, play a crucial role in the modulation of the immune response, positioning them as promising treatment options for NSCLC. Additionally, the identification of other novel targets holds the potential for addressing resistance mechanisms that are frequently encountered with current ICIs. The rapid evolution of these therapeutic options renders the selection of an appropriate treatment regimen for advanced NSCLC a complex and intricate process. It is imperative for physicians to thoroughly evaluate patient‐specific characteristics prior to initiating treatment, basing their decisions on factors such as PD‐L1 expression levels, TMB status, histological subtype, and existing comorbidities. Notwithstanding the advent of new pharmacological agents and the encouraging outcomes linked to novel targets in advanced NSCLC, continued research and refinement of treatment strategies remain essential to enhance survival outcomes within this patient demographic.

## Author Contributions

**Xingxiang Pu:** conceptualization, supervision, writing – review and editing. **Yu Zhou:** conceptualization, supervision, writing – review and editing. **Jingyi Wang:** conceptualization, supervision, writing – review and editing. **Lin Wu:** conceptualization, supervision, writing – review and editing.

## Conflicts of Interest

The authors declare no conflicts of interest.

## Data Availability

No new data were generated or analyzed in support of this research.
